# Strengthening health systems through NTD integration: Key insights from a multi-country webinar

**DOI:** 10.1371/journal.pntd.0013628

**Published:** 2026-02-17

**Authors:** Nadia Rozendaal, Marie Adama Bassabi-Alladji, Tesfahun Bishaw, Wyckliff Omondi, Aina Faes Tolotra, Salissou Adamou Batchiri, Ladislas Nshimiyimana, Piham Gnossike, Clarer Jones, Alfred Mubangizi, Maria Rebollo

**Affiliations:** 1 World Health Organization, Secretariat of the Global Onchocerciasis Network for Elimination (GONE), Geneva, Switzerland; 2 Ministry of Public Health, National NTD Programme, Porto-Novo, Benin; 3 Ministry of Health, Neglected Tropical Diseases Programme, Addis Ababa, Ethiopia; 4 Ministry of Health, Division of Vector Borne and Neglected Tropical Diseases, Nairobi, Kenya; 5 Ministry of Public Health, National NTD Control Programme, Antananarivo, Madagascar; 6 Ministry of Public Health, National NTD Programme, Niamey, Niger; 7 Ministry of Health, Rwanda Biomedical Centre—NTD Programme, Kigali, Rwanda; 8 Ministry of Health and Public Hygiene, National NTD Programme, Lomé, Togo; 9 Ministry of Health, National NTD Programme, Dodoma, United Republic of Tanzania; 10 Ministry of Health, Neglected Tropical Diseases Control Programme, Kampala, Uganda; 11 World Health Organization, Malaria and Neglected Tropical Diseases Department, Geneva, Switzerland; University of Notre Dame, UNITED STATES OF AMERICA

## Abstract

The integration of Neglected Tropical Disease (NTD) control and elimination into national health systems is a strategic imperative, driven by shifting global health priorities and evolving funding landscapes. A webinar hosted by the Global Onchocerciasis Network for Elimination (GONE) taken place on 8 July 2025 synthesizes nine country experiences demonstrating innovative models for embedding NTD interventions within established platforms, ranging from integrating mass drug administration (MDA) into maternal and child health weeks in Rwanda to leveraging national polio vaccination campaigns in Madagascar. These integrated models yielded significant, quantifiable benefits, including enhanced operational efficiency and greater national ownership. Evidence from Rwanda demonstrates the potential for achieving treatment coverage above 90% and 100% geographical coverage. Moreover, integration produced substantial cost savings, with Madagascar’s integration of MDA with polio vaccination reducing expenses by up to 89%. However, implementation is not without challenges. Common obstacles include increased workloads for health workers, gaps in health information systems, which often lack specific indicators for tracking MDA activities, and the persistent need for sustained domestic resource mobilization. Despite these hurdles, the webinar concludes that strategic, well-planned integration is a vital pathway for amplifying the impact of NTD programs. This approach not only strengthens the resilience of national health systems but also contributes meaningfully to the advancement of universal health coverage for all.

## 1. Introduction: The strategic imperative for NTD integration

The World Health Organization’s (WHO) NTD road map defines sustainability as maintaining and expanding programme progress through: (i) strengthening national health systems, (ii) improving efficiency and equitable access to treatment and care, and (iii) mainstreaming NTDs into broader health and development agendas. It emphasizes long-term domestic financing, programmatic integration, and adaptive learning to refine strategies for sustainable impact [1–4].

Amidst a landscape of contracting donor funds and the urgent pursuit of Universal Health Coverage (UHC), the integration of NTD programs has transitioned from a pragmatic option to a strategic necessity for building resilient national health systems. This evolution presents a powerful opportunity to embed NTD control and elimination into the core of national health strategies, ensuring services are more sustainable, equitable, and effective for the communities they serve.

The fundamental rationale for integrating NTD interventions with existing health platforms is clear and compelling. By aligning with primary health care, maternal and child health weeks, and national immunization and nutrition campaigns, countries can optimize scarce resources, eliminate the inefficiencies of duplicative, siloed programs, and enhance national ownership of public health initiatives. This approach moves beyond episodic campaigns to build a foundation for continuous care, strengthening the health system as a whole and bringing essential services closer to the people who need them most.

The webinar synthesizes the rich experiences and critical lessons learned from nine African nations that are pioneering diverse pathways toward NTD integration. It presents proven models for integrated service delivery, analyzes the common enablers that facilitate success, identifies the key challenges that must be overcome, and concludes with a set of actionable recommendations for national policymakers and global health partners.

The following analysis of specific country experiences provides invaluable real-world insights into building stronger, more equitable, and more resilient health systems for all.

## 2. A synthesis of successful integration models and key enablers

While the goal of NTD integration is universal, the pathways to achieving it are diverse and tailored to national and local contexts. The experiences of several African nations reveal two primary, highly effective models: leveraging existing campaign-based platforms for mass reach and embedding NTD services into routine health systems for long-term sustainability. This section analyzes these primary models, highlighting the specific strategies and key enablers that have driven their success.

### 2.1 Leveraging campaign-based platforms for mass reach and efficiency

Countries have demonstrated remarkable success in embedding NTD interventions within existing, time-bound campaigns, capitalizing on established infrastructure and community trust to achieve high coverage and significant cost efficiencies.

Madagascar: By integrating MDA for lymphatic filariasis (LF), schistosomiasis (SCH), and soil-transmitted helminthiases (STH) with national polio vaccination and vitamin A supplementation campaigns, the country achieved exceptional treatment coverage of 82% for LF and 91% for SCH/STH, with substantial cost savings of up to 89% [5].

Uganda: The integration of MDA into national Child Health Days proved highly effective, saving an estimated 10 million tablets by streamlining interventions for the same target population. This approach also significantly enhanced national ownership of the MDA activities [6,7].

Rwanda: Leveraging the established maternal and child health (MCH) week for STH and SCH MDA resulted in sustained high treatment coverage of over 90%. Critically, this model enabled the mobilization of 100% domestic resources for all operational costs and successfully engaged a wide range of multisectoral stakeholders [8,9].

United Republic of Tanzania: A pilot program integrating NTD and nutrition campaigns demonstrated the feasibility of dual delivery. The campaign successfully administered treatments for trachoma and STH alongside vital nutrition interventions like Vitamin A supplementation and mid-upper arm circumference screening [10].

### 2.2 Embedding NTD services into routine health systems for sustainability

The second major strategy involves weaving NTD services into the permanent fabric of the national health system, ensuring continuous care and building long-term institutional capacity.

Primary health care (PHC) and community health systems: Kenya has embedded NTD diagnosis and treatment into routine PHC services and empowered over 6,000 Community Health Promoters with a standardized NTD curriculum. Similarly, Benin’s model for onchocerciasis relies on community health workers (“*relais Communautaire*”), each responsible for MDA delivery within approximately 200 assigned households, ensuring deep community penetration [11].

Health information systems (HMIS): To ensure robust data for decision-making, Rwanda and Ethiopia have successfully integrated NTD data reporting into their national HMIS (DHIS2). Kenya has likewise embedded NTD indicators into its national Kenya Health Information System (KHIS) and its electronic Community Health Information System (eCHIS), creating a seamless data flow from the community to the national level [12].

School-based platforms: Benin employs a dual-platform approach for SCH and STH treatment. Teachers are trained to administer medicines in schools, while community health workers are mobilized to reach out-of-school children, with joint supervision by the Ministries of Health and Education ensuring comprehensive coverage.

Multisectoral governance: Togo provides a powerful example of structural integration. In 2018, it merged multiple vertical NTD programs into a single, unified national program. This was followed in 2022 by the creation of the National Multisectoral Coordination Framework (CNCM-MTN), a high-level body that includes representatives from 12 different ministries to ensure a coordinated, whole-of-government response [13].

The successful application of these distinct yet complementary models demonstrates a strategic shift, yielding tangible benefits that not only advance disease control but also fundamentally strengthen the underlying health system architecture.

## 3. Cross-cutting benefits: Building value beyond NTD control

The value of integrating NTD services extends far beyond disease-specific outcomes. These strategies create powerful synergistic benefits that strengthen the entire health system, enhance program sustainability, and generate significant returns on investment. The evidence from across the continent demonstrates clear, quantifiable gains in efficiency, systemic capacity, and national ownership.

**Financial and resource efficiency:** By sharing logistics, supervision, and community mobilization efforts, integrated models achieve substantial cost savings. In Benin, integration reduced costs by 50% for onchocerciasis and an impressive 75% for SCH/STH programs. Madagascar saved over US$1.4 million in a single integrated campaign. Furthermore, Rwanda successfully mobilized 100% of its operational costs from domestic resources for STH and SCH MDA conducted through MCH week, demonstrating a clear pathway to financial sustainability.

**Health system strengthening:** More than a set of disparate activities, integration serves a single strategic goal: institutionalizing NTD control within the permanent structures of the primary healthcare system. The training of thousands of frontline health workers in Kenya (over 6,000) and Rwanda (over 400 in a recent pilot) creates a versatile and skilled workforce. The inclusion of NTD indicators into national data systems (HMIS/DHIS2) in Ethiopia and Kenya enhances routine disease surveillance. Concurrently, Ethiopia has integrated NTD medicines into its national supply chain, a core component of the PHC integration strategy being pursued by countries like Kenya, which is now domestically procuring essential NTD medicines [12,14,15]. These actions embed NTD control into the core functions of the health system.

**Enhanced program sustainability and ownership:** Moving away from partner-led vertical programs fosters greater national leadership and long-term viability. Togo’s experience confirms that integration enhances national ownership of its NTD program. This sentiment is echoed in Uganda, where officials observed that MDA activities are now more firmly embedded within the national health system rather than being perceived as externally driven initiatives.

While these benefits confirm the strategic value of integration, realizing this potential at scale requires a clear-eyed assessment and mitigation of the persistent obstacles to implementation.

## 4. Overcoming implementation challenges

Despite the clear benefits across countries, integrated NTD programmes are making progress but continue to face shared implementation challenges that present opportunities for strengthening. Enhanced coordination and governance are priorities in Benin, Niger [16], and Togo, while investments in the health workforce would benefit Ethiopia, Kenya, Uganda, Madagascar, Rwanda, and the United Republic of Tanzania. Sustainable financing and increased domestic resource mobilization remain important in Ethiopia, Kenya, Rwanda, and Togo. Strengthening digital tools, data systems, and logistics would further support programmes in Ethiopia, Kenya, Uganda, Rwanda, Madagascar, and Tanzania, while improved communication and targeted strategies could help address treatment acceptance and coverage gaps, particularly in Madagascar and Uganda. Remaining hurdles, far from being deterrents, provide a clear and evidence-based roadmap for the targeted policy actions and strategic investments required to scale and sustain NTD integration.

As summarized in Table 1, the countries adopted different integration approaches based on health-system capacity.

**Table 1:** Summary of NTD integration strategies by country.

Country	Integration model	Delivery platform	Key outcomes	Notable challenges
Benin	• School and community-based MDAs	• Community health workers for onchocerciasis • Schools and community workers	• 50% cost reduction for onchocerciasis • Up to 75% savings for SCH/STH • Improved coverage and sustainability	• Need for stronger coordination • Capacity-building for sustainability
Ethiopia	• Integrated into decentralized primary health-care system;	• Health extension workers, teachers • Health Development Army • Electronic Collaborative Action Strategy piloted in 2024 for campaign coordination	• Millions reached via MDA and surgery • 5 Integrated campaigns (e.g., HPV, polio) • Inclusion of NTDs in national health information system	• HR shortages • Financing gaps • Digital integration needs • Logistical constraints
Kenya	• Government-led integration into primary health care	• Community health promoters (CHP) and electronic Community Health Information System used	• 6000 CHPs and 400 assistants trained • Increased coverage • Reduced patient costs • Improved data flow • Stronger governance	• Health worker overload • Limited MDA-specific data tools • Lack of sustained funding and policy support
Madagascar	• MDA + polio + vitamin A campaign	• Door-to-door and fixed-point strategies	• 90% coverage, $1.4M saved • 82% treatment coverage • 100% geographical coverage • Up to 89% cost savings	• Staff workload • Supply chain complexity • Unclear messaging • Minor adverse events and some treatment refusals
Niger	• Since 2007 annual integrated MDA campaigns with unified teams and shared logistics	• Unified national teams • Shared planning and logistics	• Elimination of onchocerciasis transmission • Control of LF • Effective management of trachoma and intestinal worms • Improved geographical and therapeutic coverage	• Sustaining large-scale coordination • Need for adaptive management and continuous strategic planning
Rwanda	• Multisectoral NTD integration within health and across ministries; -SCH/STH MDA integrated into maternal and child health weeks • Cross-training entomologists for mosquitoes, snails, and black flies. • Transition strategy to sustain key MCH week campaign in routine care.	• Community health workers • Schoolteachers • Rwanda Biomedical Centre coordination • Partners	• Achieved >90% treatment coverage • 100% geographical coverage for STH/SCH • Since 2021, adults included in all MDA rounds • 100% operational costs funded domestically • Integrated Skin NTD screening and management pilot in 4 districts	• Financing and staffing constraints • Digitalization demands investment
Togo	• National Multisectoral Coordination Framework for NTDs in 2022, operational in 2023 (incl 12 Ministries)	• Pilot integrated MDA SCH/STH maritime region	• Enhanced coordination and cost-effectiveness • Stronger national ownership	• Need for flexible external support • Lack of fully functional structure • Mobilizing domestic funding to reduce external dependency
Uganda	• All case management NTDs (e.g., kala azar, HAT, scabies, rabies) integrated into primary health care. • MDA for PC-NTDs integrated into Child Health Days, targeting children <15 years and pregnant women.	• Joint planning with Uganda National Expanded Program on Immunization • District focal persons • Primary health facilities	• Nationwide coverage • 10M tablets saved • Strengthened system capacity	• Health worker overload • Limitations of Health Management Information system • Coverage gaps for preschoolers
United Republic of Tanzania	• Integrated MDA with nutrition services; pilot in co-endemic districts for trachoma and STH	• House-to-house and fixed-point • Community and district coordination	• Improved reach and efficiency • Progress in NTD and nutrition outcomes	• Complex training and logistics • Staff turnover due to delayed incentives

**Fig 1:**
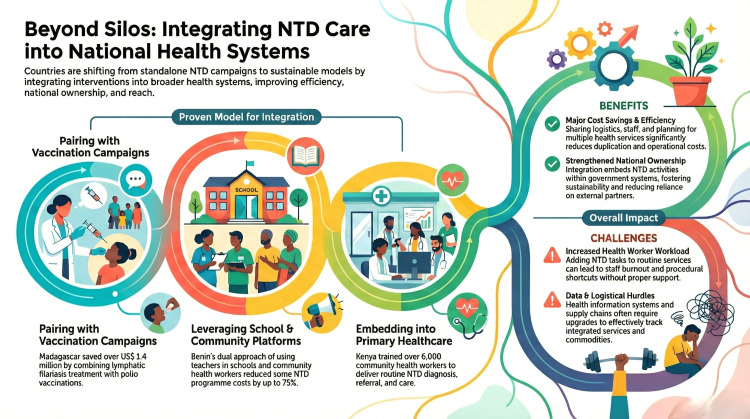
Beyond silos: integrating NTD care into national health systems. Diagram illustrating approaches for integrating NTD interventions into broader health systems: pairing with vaccination campaigns, leveraging school and community platforms, and embedding NTD services into primary healthcare. Image credit: Nadia Rozendaal.

As shown in Fig 1, approaches for integrating NTD care vary across delivery platforms.

## 5. Policy recommendations

The collective experiences of African nations demonstrate that, while complex, integration of NTD programs delivers substantial benefits for individuals, health systems, and national development. Integrated approaches reduce duplication, enable people to access multiple services in a single visit, and strengthen national ownership. Ultimately, integration is a vital pathway for amplifying public health impact and accelerating progress toward UHC.

### 5.1 Core policy recommendations

Promote domestic and flexible financing: Increase national budget allocations for NTDs and their integration into primary health care, following Rwanda and Kenya’s leadership. Donors should complement this by providing flexible, long-term funding aligned with integrated national strategies rather than disease-specific priorities.

**Foster a culture of learning and adaptation:** Support platforms for cross-country learning, such as the GONE webinar series, to share best practices and innovations. Examples include Madagascar’s cost-effective campaign integration and Rwanda’s use of digital health tools for community-based care. Expanding opportunities for experience-sharing with countries not represented in this review—through targeted bilateral exchanges, regional meetings, and dedicated publications—would help translate successful approaches across contexts, accelerate adaptive programming, and strengthen the resilience and effectiveness of national NTD programmes.

**Institutionalize multisectoral coordination:** Establish formal, high-level coordination bodies—such as Togo’s framework—to ensure policy alignment, resource sharing, and joint accountability across ministries of health, education, finance, and water and sanitation. This embeds NTD control within broader national development goals and breaks down sectoral silos.

**Invest in health systems strengthening:** Prioritize targeted investments in integrated data systems, unified supply chains, and joint supervision mechanisms. In 2024, Ethiopia became one of the first countries to pilot and adopt the Health Campaign Effectiveness Coalition (HCE) Collaborative Action Strategy (CAS) by creating its own Ethiopian CAS (or E-CAS) and pilot testing the Electronic Collaborative Action Strategy (E-CAS) to enhance campaign coordination [17]. A National Steering Committee and specialized task teams were established to guide planning, monitoring, financing, and implementation. The strategy facilitated integration of NTD services with other health campaigns, including vaccinations, malaria testing, ITN distribution, nutrition screening, and services for pregnant and lactating women.

**Empower the frontline health workforce:** Develop standardized, integrated training curricula and digital job aids, as pioneered in Kenya, to equip community health workers with the skills to deliver comprehensive services. Pair these with fair incentive structures to sustain motivation and prevent burnout.

### 5.2 Forward-looking recommendations

Champion a global community of practice: Formalize knowledge exchange through dedicated communities of practice, building on models like GONE and WHO’s regional learning initiatives. These platforms should disseminate harmonized tools, curricula, and strategies for overcoming common barriers.

**Economic Modeling to Inform NTD Investment:** There is a need for robust modeling and economic analyses to quantify the impact of funding reductions on national NTD budgets and intervention outcomes [18]. Mandating cost-effectiveness and return-on-investment analyses would strengthen the evidence base for integrated NTD programming. While data from Madagascar and Benin show substantial cost savings, broader analyses are needed to inform resource allocation and support sustained domestic and donor investment.

**Develop financial mitigation strategies:** Safeguard elimination targets by planning for funding volatility. Rwanda’s domestic financing of MDA costs and Kenya’s inclusion of NTDs in social health insurance provide replicable models for resilience.

**Align global and regional support with country-led priorities:** Ensure that WHO and donor initiatives remain flexible and responsive to national strategies, such as Ethiopia’s Collaborative Action Strategy. Multisectoral governance structures in Togo, Kenya, and Rwanda illustrate how country-led frameworks can anchor sustainable integration.

## 6. Conclusion: Integration as a cornerstone of UHC

NTD integration is not merely an operational efficiency measure—it is a strategic approach that strengthens resilience, amplifies impact, and fosters national ownership. With thoughtful planning, sustained investment, and collaborative innovation, integration can accelerate progress toward UHC and health equity. The evidence is clear, the models are proven, and the time for siloed approaches has passed. Building on these lessons, stakeholders can forge pathways that eliminate neglected diseases while creating stronger, more equitable health systems for all.
